# Work ability following breast cancer – the MyHealth randomized controlled trial

**DOI:** 10.2340/1651-226X.2025.42221

**Published:** 2025-01-08

**Authors:** Trine A. Horsbøl, Lena Saltbæk, Caroline Urhammer, Randi V. Karlsen, Christoffer Johansen, Pernille E. Bidstrup, Beverley L. Høeg, Vibeke Zoffmann, Federica Belmonte, Ingelise Andersen, Anne S. Friberg, Mads N. Svendsen, Helle G. Christensen, Vesna Glavicic, Dorte L. Nielsen, Susanne O. Dalton

**Affiliations:** a National Institute of Public Health, University of Southern Denmark, Copenhagen, Denmark; b Cancer Survivorship, Danish Cancer Institute, Copenhagen, Denmark; c Department of Clinical Oncology and Palliative Care, Zealand University Hospital, Denmark; d Institute of Clinical Medicine, Faculty of Health, Copenhagen University, Copenhagen, Denmark; e Department of Oncology, CASTLE, Copenhagen University Hospital, Copenhagen, Denmark; f Psychological Aspects of Cancer, Cancer Survivorship, Danish Cancer Institute, Copenhagen, Denmark; g Institute of Psychology, Faculty of Social Sciences, Copenhagen University, Copenhagen, Denmark; h Research Unit of Women’s and Children’s Health, the Juliane Marie Center, Copenhagen University Hospital, Copenhagen, Denmark; i Statistics and Data Analysis, Danish Cancer Institute, Copenhagen, Denmark; j Section of Social Medicine, Department of Public Health, Faculty of Health and Medical Sciences, University of Copenhagen, Denmark; k Department of Oncology, Herlev and Gentofte University Hospital, Herlev, Denmark

**Keywords:** Randomized controlled trial, work ability, breast cancer

## Abstract

**Background and purpose:**

We previously demonstrated positive effects on quality of life and mental health following breast cancer when comparing a nurse-led follow-up program without scheduled visits (MyHealth) to regular follow-up. This study aims to examine whether MyHealth also positively impacts self-reported work ability.

**Patients/material and methods:**

A total of 288 patients, potentially active on the labour market, were randomized to MyHealth or control follow-up after primary treatment for early-stage breast cancer (2017–2019). MyHealth included individual self-management sessions, electronic symptom monitoring, and assistance with navigating healthcare services. Control follow-up consisted of biannual outpatient visits with a physician. Linear mixed-effect models were applied to evaluate the effect of MyHealth on self-reported work ability at 6, 12, 24, and 36 months after randomization as measured by the Work Ability Score (WAS).

**Results:**

Work ability increased significantly in both groups during the first 6 months (mean WAS increase MyHealth: 1.64, 95% confidence interval [CI]: 1.26; 2.02 and control: 1.57, 95% CI: 1.17; 1.97) and continued to increase slightly but non-significantly (*p*-values > 0.13) until end of follow-up at 36 months. Improvement was especially pronounced among patients reporting poor work ability at baseline. Differences in mean WAS between patients in MyHealth and control follow-up were non-significant and close to zero at all time points (–0.21 to 0.48).

**Interpretation:**

The MyHealth follow-up program had no additional effect on self-reported work ability compared to regular follow-up. Future interventions should target patients with poor work ability and include components specifically designed to enhance work ability.

## Introduction

In Nordic countries, 21,640 women are diagnosed with breast cancer yearly, with almost half being 25–64 of age, and likely to be occupationally active [1]. For many, maintaining or resuming employment is vital [2], motivated by the need for financial stability and a sense of normalcy [3]. Thus, it is crucial to provide these women with support to maintain or regain their work ability.

Findings from a meta-analysis show that 70% (95% confidence interval [CI]: 69–82%) of women return to work within 2 years after a breast cancer diagnosis [4]. However, women who have returned to work, have more sick leave [5] and report poorer work ability than the general population [6–8]. Work ability is a multidimensional concept encompassing a worker’s physical, psychological, and social capacity to manage job demands [9]. Reduced work ability after breast cancer is often attributed to physical or psychosocial challenges [10, 11].

We lack evidence-based strategies to support breast cancer survivors’ work ability [12, 13]. A recent systematic review on interventions to support return to work among patients with breast cancer identified nine randomized controlled trials (RCTs) [14], of which only one intervention proved effective. This intervention comprised a 2-week multicomponent program with physical and nutritional elements, which led to increased self-reported work ability at 12 months of follow-up [15].

We conducted an RCT assessing the effect of a nurse-led follow-up program including self-management sessions and electronic symptom monitoring without scheduled visits (MyHealth) compared to scheduled outpatient visits with physicians following primary breast cancer treatment. As recently reported, MyHealth significantly improved health-related quality of life and reduced fear of recurrence, anxiety, and depression through 3 years of follow-up [16]. The MyHealth follow-up program did not have a specific occupational focus, but we hypothesize that the positive effect on quality of life and mental health may subsequently improve self-reported work ability, as this association has been established in previous research [10, 11]. Thus, in this study, we report results on self-reported work ability as a secondary outcome in the MyHealth study.

## Patients/material and methods

MyHealth is a randomized two-group parallel trial comparing nurse-led individualized follow-up to regular physician-led follow-up conducted at the Department of Clinical Oncology and Palliative Care, Zealand University Hospital, Denmark [16, 17].

### Participants and randomization

All consecutive patients were screened for eligibility over a period spanning January 2017–January 2019. Eligible patients were women, at least 40 years old, who had completed primary treatment with curative intent for stage I or II breast cancer within 2 months, who scored 0–3 in Eastern Cooperative Oncology Group (ECOG) performance status and were able to understand and speak Danish. Exclusion criteria were recurrent breast cancer, residual disease, genetic predisposition for breast cancer, presence of other active cancers except non-melanoma skin cancer, severe cognitive impairment or psychiatric disease, or addiction to alcohol or narcotics.

In all, 503 patients were randomized either to MyHealth or control follow-up using an electronic platform that secured concealed allocation. In the present study, we only included participants who were potentially active at the labour market at randomization. Thus, we excluded patients who had retired due to age (*n* = 150) or were granted disability pension or flexi job due to permanently reduced work ability (*n* = 35). We also excluded patients aged 65 years or older (*n* = 26), as we assumed that they would retire due to age during follow-up. Lastly, we excluded patients with missing values on work status or work ability (*n* = 4). This left 149 patients to MyHealth and 139 to control follow-up for this analysis.

### MyHealth follow-up

The MyHealth follow-up program included a self-management intervention, regular symptom reporting, and support by experienced breast cancer nurses who had completed a 6-day course covering the Guided Self-Determination (GDS) method, breast cancer late effects, recurrence symptoms, and navigation [16, 17]. MyHealth consisted of three to five 1-hour individual sessions during a 6-month period after randomization following the GDS method, which aims to empower patients in decision-making and problem-solving [18]. The sessions were structured by reflection sheets to identify the patient’s challenges and improve self-management strategies. Further, the patients reported symptoms electronically every third months the first year, and every sixth months the following 2 years, which were monitored by the nurses to identify symptoms of late effects or potential breast cancer recurrence. The electronic questionnaire included the 23-item Breast Cancer Recurrence instrument [19] and 19 items on adverse or late effects of cancer treatment [16, 17]. If the symptoms exceeded predefined thresholds, the nurses would contact the patient to clarify the need for support or referral to further evaluation or appropriate medical care. Patients could also directly contact the nurses by phone if needed [16, 17].

### Control follow-up

Patients in the control group received biannual consultations with physicians at the oncology outpatient clinic, including physical examination and unstructured symptom assessment to screen for breast cancer recurrence and late effects. If needed, the patients could request extra consultations [16, 17].

### Work ability

Self-reported work ability was measured at baseline (randomization), and after 6, 12, 24, and 36 months using the single-item Work Ability Score (WAS) [20, 21] from the Work Ability Index [22], which has been found valid to assess status and progress of work ability. Respondents are asked to evaluate their ‘current work ability compared with the lifetime best’ through a score on a scale of 0 (‘completely unable to work’) to 10 (‘work ability at its best’).

We utilized both a continuous and categorical measure of work ability (poor: 0–5; moderate to excellent: 6–10) [9].

### Statistics

A linear mixed model was used to estimate the effect of MyHealth follow-up on work ability. To assess the effect of MyHealth follow-up over time, the model included an interaction between randomization group and time (baseline, and after 6, 12, 24, and 36 months), assuming that there was no difference between the groups at baseline [23]. The model included a random intercept for each patient. Missing data on work ability were assumed to be missing at random. The analyses were conducted on the entire population, and separately among patients with poor (WAS 0–5) and moderate to excellent work ability (WAS 6–10). The analyses were performed using Stata version 18.

## Results

At baseline, the majority of the 288 patients were working (89% in MyHealth and 90% in control follow-up), while the remaining patients were either on sick leave or unemployed (Table 1). Among those allocated to MyHealth follow-up, a total of 43% reported poor work ability (WAS, 0–5), as did 39% in the control group while the median WAS was 6 in both groups (Table 1).

**Table 1:** Baseline characteristics.

	MyHealth follow-up		Control follow-up	
	No. 149	%	No. 139	%
**Age**				
40–55 years	71	48	74	53
56–65 years	78	52	65	47
**Cohabitation**				
Yes	106	71	115	83
No	43	29	24	17
**Education**				
Primary and lower secondary school (9–10 years)	15	10	16	12
Senior high school and vocational education (10–12 years)	21	14	23	17
University level 1–2 years 12–15 years)	26	17	33	24
University level ≥ 3 years (≥15 years)	87	58	67	48
**Employment**				
Employed/self-employed	133	89	125	90
On sick leave	10	7	8	6
Unemployed	6	4	6	4
**Menopausal status**				
Premenopausal	56	38	52	37
Postmenopausal	93	62	87	63
**Tumour size**				
<21 mm	115	77	118	85
21–49 mm	34	23	21	15
**Histology**				
Ductal	120	81	119	86
Lobular	17	11	12	9
Other	12	8	8	6
**Grade of malignancy**				
I	27	18	26	19
II	76	51	73	53
III	32	21	29	21
Not relevant^1^ or unknown	14	9	11	8
**Oestrogen receptor status**				
Positive (≥1%)	137	92	124	89
Negative	12	8	15	11
**HER2 status**				
Amplified	20	13	14	10
Not amplified	129	87	125	90
**Number of lymph nodes with macro metastases**				
No lymph nodes	119	80	106	76
1–3 lymph nodes	22	15	30	22
>3 lymph nodes	8	5	3	2
**Breast surgery**				
Lumpectomy	128	86	118	85
Mastectomy	21	14	21	15
**Axillary surgery**				
Sentinel node biopsy	121	81	106	76
Axillary dissection	28	19	33	24
**(Neo-) Adjuvant chemotherapy**				
Yes	98	66	87	63
No	51	34	52	37
**Adjuvant radiotherapy**				
Yes	132	89	126	91
No	17	11	13	9
**Adjuvant endocrine therapy**				
Yes	132	89	120	86
No	17	11	19	14
**Adjuvant trastuzumab**				
Yes	20	13	14	10
No	129	87	125	90
**Work ability (WAS)**				
Poor (0–5)	64	43	54	39
Moderate (6–7)	35	23	34	24
Good (8–9)	42	28	40	29
Excellent (10)	8	5	11	8

HER2: Human epidermal growth factor receptor 2.

1 Grade of malignancy not relevant as histology is neither ductal nor lobular.

**Figure 1:**
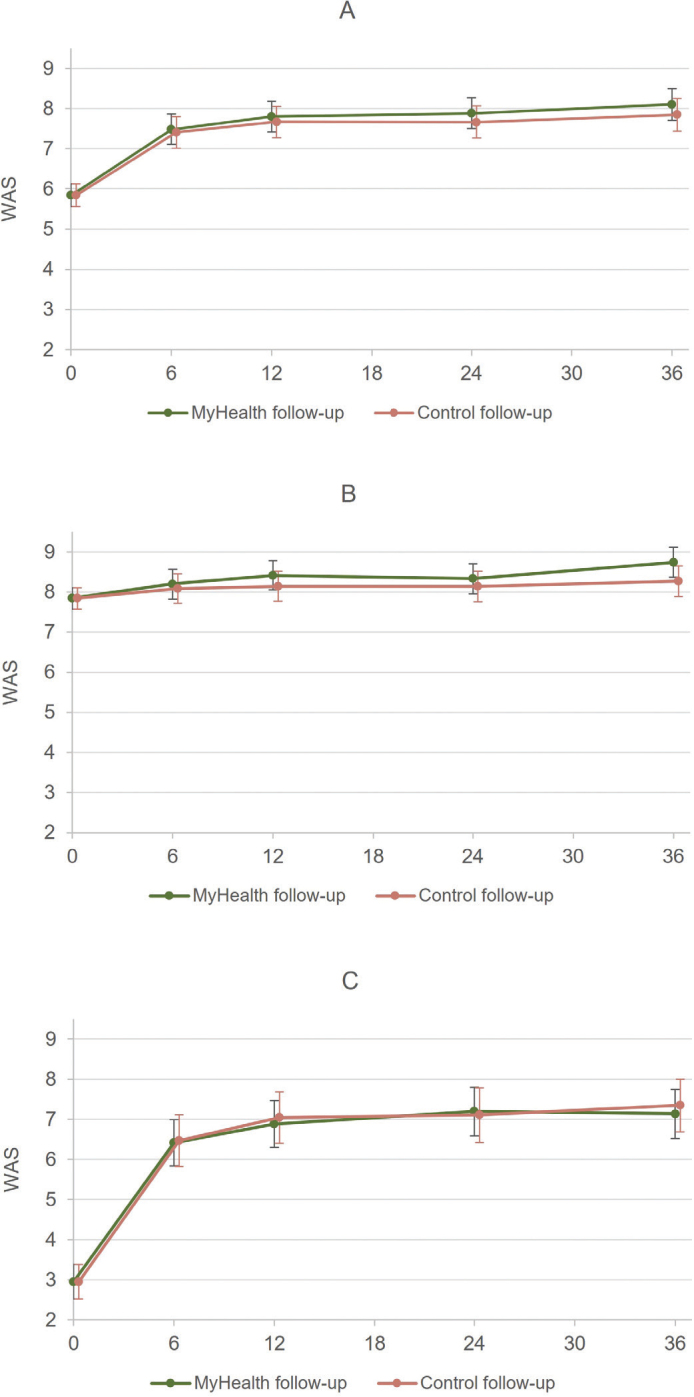
(A) Levels of work ability for all patients in MyHealth and control follow-up at baseline (randomization) and at 6, 12, 24, and 36 months of follow-up. (B) Levels of work ability for patients in MyHealth and control follow-up with initial moderate, good, or excellent work ability (WAS 6–10) at baseline and at 6, 12, 24, and 36 months of follow-up. (C) Levels of work ability for patients in MyHealth and control follow-up with initial poor work ability (WAS 0–5) at baseline and at 6, 12, 24 and 36 months of follow-up.

For both groups, WAS increased significantly during the first 6 months following randomization (mean WAS increase MyHealth: 1.64, 95% CI: 1.26; 2.02 and control: 1.57, 95% CI: 1.17; 1.97) (Table 2), and continued to increase slightly but non-significantly (*p*-values > 0.13) until end of follow-up at 36 months, where it reached a mean score of 8.09, 95% CI: 7.71; 8.49 for patients in MyHealth follow-up and 7.85, 95% CI: 7.44; 8.25 for those in control follow-up (Figure 1A). Improvements in work ability were most pronounced among patients reporting poor work ability at baseline, with WAS increasing from less than 3 to more than 7 during follow-up for patients in both MyHealth and control follow-up (Figure 1C, Table 2).

**Table 2:** Difference in work ability score from baseline (randomization) to 6, 12, 24, and 36 months of follow-up for patients in intervention follow-up compared to control follow-up.

Time (months)	N_MyHealth_ / N_Control_	Mean WAS at baseline	Mean difference in WAS from time 0 (baseline)				Difference between MyHealth and control follow-up	
			MyHealth		Control			
			Mean WAS difference	95% CI	Mean WAS difference	95% CI	Mean WAS difference	95% CI
**0**	149/139	5.84						
**6**	142/127		1.64	1.26; 2.02	1.57	1.17; 1.97	0.07	–0.44; 0.58
**12**	141/125		1.96	1.58; 2.34	1.83	1.43; 2.23	0.13	–0.38; 0.64
**24**	130/117		2.04	1.65; 2.44	1.82	1.41; 2.23	0.22	–0.31; 0.75
**36**	128/117		2.26	1.86; 2.65	2.01	1.59; 2.42	0.25	–0.28; 0.78
**Patients with moderate, good, or excellent work ability (WAS 6–10) at baseline**								
**0**	85/85	7.85						
**6**	81/78		0.35	–0.02; 0.73	0.25	–0.13; 0.63	0.10	–0.39; 0.60
**12**	82/76		0.57	0.19; 0.94	0.30	–0.09; 0.68	0.27	–0.22; 0.76
**24**	76/75		0.49	0.11; 0.87	0.29	–0.10; 0.68	0.20	–0.30; 0.70
**36**	74/72		0.90	0.51; 1.29	0.42	0.03; 0.81	0.48	–0.03; 0.99
**Patients with poor work ability (WAS 0–5) at baseline**								
**0**	64/54	2.95						
**6**	61/49		3.47	2.85; 4.08	3.52	2.85; 4.19	–0.05	–0.88; 0.77
**12**	59/49		3.93	3.31; 4.55	4.10	3.43; 4.77	–0.17	–1.00; 0.66
**24**	54/42		4.25	3.61; 4.89	4.16	3.45; 4.87	0.09	–0.79; 0.97
**36**	54/45		4.19	3.55; 4.83	4.40	3.70; 5.09	–0.21	–1.07; 0.66

CI: confidence interval; WAS: Work Ability Score.

### Effect of intervention

We did not find improved work ability among the patients in MyHealth follow-up compared to the control group at six, 12, 24, and 36 months after randomization (Figure 1A, Table 2). Differences in mean WAS between the two groups were close to zero at all time points (0.07–0.25) and were not statistically significant (Table 2). This was also the case, when we stratified the population on poor and moderate to excellent work ability at randomization (Figure 1B-C, Table 2).

## Discussion

Work ability improved significantly over time in both follow-up programs, especially among patients with poor work ability at randomization. The improvements were similar in the MyHealth and control follow-up, and thus MyHealth had no additional effect on self-reported work ability compared to regular follow-up.

It is encouraging that patients in both follow-up programs experienced a rapid improvement in self-reported work ability following primary breast cancer treatment. Patients in both groups achieved what is considered moderate to good work ability (mean WAS 8.1/7.9), approaching levels comparable to those of female general populations (mean WAS 8.2–8.9) [6–9]. Self-reported work ability among patients with breast cancer varies across Scandinavian studies (mean WAS 6.3–8.7) [6, 7, 24]. Comparisons to our results are challenged by a lack of prospective studies, differences in time since diagnosis, and as the study populations vary in age and stage of disease [6, 7, 24].

Previous studies suggest that interventions to improve work ability should specifically target patients with poor work ability [13]. One third of the population in this study reported good to excellent work ability already at randomization and were thus not subject to considerable improvements. When we examined the effects of MyHealth separately for patients with poor and moderate to excellent work ability, no effect on work ability was seen in either of the groups. Still, it is noteworthy that work ability increased considerably during follow-up for those with poor work ability at randomization (from WAS less than 3 to more than 7). Although they did not reach the same levels as those with good to excellent work ability at randomization, they achieved a moderate level of work ability. We did not have information on work ability prior to the breast cancer diagnosis, and for some patients reporting poor work ability at randomization, their habitual work ability might already have been lower than in the general population for reasons other than breast cancer.

No previous RCTs have assessed the effect of a multicomponent follow-up program on self-reported work ability among patients with breast cancer. Furthermore, previous intervention studies tend to focus solely on return to work, number of working hours, and sick leave [14]. Emphasizing enhancement of perceived work ability, rather than solely focusing on return to work, is crucial, as cancer survivors often continue to face physical and psychosocial late effects that affect their work ability beyond return to work [10, 11].

Several studies underline the importance of including elements aimed to explicitly enhance work ability, and to involve the workplace in interventions that aim at supporting cancer patients to return to work [25–27]. The MyHealth follow-up program did not include a systematic occupational focus. The reflection sheets used to structure the sessions with nurses were designed to identify the challenges experienced by each patient. Thus, the sessions covered a range of topics, and not all women mentioned work ability as one of their concerns. The absence of an effect on work ability may be due to the lack of elements directly targeting work.

### Strengths and limitations

Internal validity in the MyHealth study is high due to the randomized design, low attrition, long follow-up, and modest amount of missing information. The main limitation is that the MyHealth follow-up program did not include a targeted occupational element. Second, one third of the population reported good to excellent work ability already at randomization and were thus not subject to considerable improvements. Third, no sample size calculation was conducted for self-reported work ability as an outcome, and this study could be under-powered. However, the differences in work ability between patients in MyHealth and control follow-up were consistently close to zero, and confidence intervals were not considerably broad. Fourth, of invited patients, 57% consented to participate. There may be systematic differences between participants and non-participants for example in terms of educational level, and the results of this study may not be generalizable to patients with low educational level. It further limits generalizability that the participants only included women aged 40–64 years old with early-stage breast cancer.

## Conclusions

The MyHealth follow-up program had no additional effect on self-reported work ability compared to regular follow-up. It is though encouraging that work ability increased rapidly over time across both groups following primary treatment.

Future interventions that aim to support patients with breast cancer in regaining work ability should be targeted those with poor work ability during or after breast cancer treatment. Also, such interventions should include elements aimed to explicitly enhance work ability.

## Data Availability

Danish legislation prohibits sharing patient data, but collaboration with other researchers is welcomed. Data can be analysed under collaborative study protocols at the Danish Cancer Institute.
